# HEWL interacts with dissipated oleic acid micelles, and decreases oleic acid cytotoxicity

**DOI:** 10.1371/journal.pone.0212648

**Published:** 2019-02-22

**Authors:** Qin Huang, Dan Sun, Muhammad Zubair Hussain, Yonggang Liu, Ludmilla A. Morozova-Roche, Ce Zhang

**Affiliations:** 1 Laboratory of Stem Cell and Tissue Engineering, Chongqing Medical University, Chongqing, China; 2 State Key Laboratory of Cultivation Base for Photoelectric Technology and Functional Materials, Institute of Photonics and Photon-Technology, Northwest University, Xi’an, China; 3 Department of Zoology, Government Emerson College, Multan, Pakistan; 4 Department of Medical Biochemistry and Biophysics, Umeå University, Umeå, Sweden; Russian Academy of Medical Sciences, RUSSIAN FEDERATION

## Abstract

Senile plaques are well-known hallmarks of Alzheimer’s Diseases (AD). However, drugs targeting tangles of the protein tau and plaques of *β*-amyloid have no significant effect on disease progression, and the studies on the underlying mechanism of AD remain in high demand. Growing evidence supports the protective role of senile plaques in local inflammation driven by S100A9. We herein demonstrate that oleic acid (OA) micelles interact with hen egg white lysozyme (HEWL) and promote its amyloid formation. Consequently, SH-SY5Y cell line and mouse neural stem cells are rescued from OA toxicity by co-aggregation of OA and HEWL. Using atomic force microscopy in combination with fluorescence microscopy, we revealed that HEWL forms round-shaped aggregates in the presence of OA micelles instead of protofibrils of HEWL alone. These HEWL amyloids act as a sink for toxic OA micelles and their co-aggregate form large clumps, suggesting a protective function in amyloid and OA cytotoxicity.

## Introduction

For decades, senile plaques and neurofibrillary tangles have been regarded as the hallmarks of Alzheimer’s Diseases (AD). The AD senile plaques are composed of amyloid fibrils of A*β* peptides. In other known to date human diseases, amyloid fibrils are result of protein misfolding and aggregation of other amyloidogenic proteins and peptides, all of which self-assemble into cross-*β*-sheet containing amyloid fibrils [1–3]. Recent studies indicate that amyloid aggregates (early and late stage of senile plaque) are not the direct cause of dementia [4–7]. The degree of cognitive impairment has only weak correlation with the quantities of aberrant amyloid plaque deposits, which are commonly found in individuals with mild or no sign of cognitive decline [8]. To date, numerous therapeutic studies and clinical trials targeting A*β* amyloid have failed [9–11]. With advanced knowledge of AD underlying mechanism, new "disease modifying" therapeutic strategies have been explored, e.g. targeting neuroinflammation and Ca^2+^ homeostasis [12, 13].

However, as the brain is an extremely complex system [14, 15], it is difficult to reveal the mechanism of brain disorder by studying any individual contributor. Effect of single component may be inverse depending on the context, e.g. the previously deemed harmful A*β* amyloids may help rescue neurons from acute inflammation. Our previous study revealed that the formation of A*β* amyloids could be a protective response, mediating pro-inflammatory S100A9 neurotoxicity [16].

Half of brain dry weight is lipids, in which fatty acids are the constituting blocks [17]. Growing evidence suggests that neurodegenerative diseases were associated with abnormal fatty acid metabolism [18–23]. Disturbances of fatty acid metabolism in the brain induce neurological disorders, which are responsible for neurodegenerative diseases such as AD [24–27]. Senile plaque formation and neurofibrillary tangle burden are found to be directly associated with the quantities of fatty acids [8]. Experiments with AD transgenic mice demonstrated a protective effect of docosahexaenoic acid (DHA)preventing brain cell death, and positive effects in the inhibition of amyloid formation [28, 29]. The results are, however, contradictory on the effect of fatty acids in inducing coma in experimental animals [30]. As a surfactant, fatty acids are reported to be able to promote amyloid formation and induce large amyloid aggregates, which could be related to senile plaques production in AD [31–33].

To address these seemly contradictory findings, we herein investigated the effect of fatty acids on neuron viability using oleic acid (OA) and hen egg white lysozyme (HEWL) as modeling molecules. Oleic acid is a fatty acid that present naturally in various animal and vegetable fat. It is toxic to Jurkat, macrophage and neuroblastoma cells [34–36]. Structurally, oleic acid has one hydrophobic and one hydrophilic end, and is used as surfactant in many studies [37]. HEWL, due to its amyloid forming capacities and low cost, is often used as an in vitro protein model [38]. The studies on HEWL and OA interaction reveal that OA as surfactant, may initiate protein aggregation and amyloid formation [31–33]. By positioning OA and HEWL mixture under extreme conditions, we observe that OA, in the form of micelles, promotes HEWL amyloid formation. HEWL amyloid self-assembly, which was monitored by Thioflavin-T assay (ThT) and circular dichroism (CD), was initiated by formation of round-shaped aggregates. During prolonged incubation, large protein aggregates (clumps) were formed. The quantity of the aggregates and their amyloid contents were proportional to OA concentration. To investigate the effect of HEWL-OA complexes on cells of neural origin, the viability of SH-SY5Y cell line and mouse neural stem cell was measured. Our results revealed that freshly dissolved HEWL and HEWL clumps were not toxic to neural cells, whereas OA micelles show strong cytotoxicity. Interestingly, OA micelle cytotoxicity was significantly reduced after incubation with HEWL. Thus, our study provides the evidence that excessive OA (over micelle critical concentration) promotes the formation of large HEWL aggregates, which implies that amyloid plaque formation is related to fatty acids and may be the result of a natural protective response.

## Materials and methods

### Protein samples

HEWL (Aldrich-Sigma) was used without any further purification. All HEWL samples were dissolved in 20 mM glycine buffer, and adjusted to pH 2.3 before mixing with OA. The protein concentration was determined by weight and NanoDrop measurements. Mixtures of HEWL, OA and dimethyl sulfoxide (DMSO) are named after their contents: P (1.4 mM HEWL); PD (1.4 mM HEWL and 5.6 M DMSO); POA10 (1.4 mM HEWL and 14 mM OA); POA100 (1.4 mM HEWL and 140 mM OA). In order to produce and maintain OA micelles, all samples were subjected to continuous shaking at 800 rpm and 57°C. All chemicals were purchased from Sigma unless mentioned differently.

### Amyloid kinetics assay

In amyloid kinetic assay we have measured ThT fluorescence, which increases when dye binds selectively to amyloids. A ThT stock solution was made by dissolving 2.5 mM ThT (Merck-Schuchardt) in phosphate buffer (10 mM phosphate, 150 mM NaCl, pH 7.4) and filtered before use. This stock solution was diluted 50-fold in the phosphate buffer to produce the working solution. 10 *μ*l of protein sample was added to 300 *μ*l of the working ThT solution, and was allowed to bind to ThT for 1 min. ThT fluorescence was measured using a FluoroMax-2 spectrophotometer (Jobin Yvon/Psex Instruments) with excitation and emission wavelengths of 440 and 485 nm, respectively, and a slit width of 5nm. The ThT fluorescence intensities were normalized to the fluorescence of the free dye in solution.

### CD Spectroscopy

CD spectra were recorded using a JASCO J-720 spectropolarimeter equipped with a PTC-343 temperature controller. For each sample, 3 spectra were acquired and averaged, using the spectral range of 190 nm to 250 nm, a 5 nm/min scan speed, and 1 nm resolution. The quartz cells had a 1 mm optical path. The contribution from the buffer was subtracted, and the results are presented in relative ellipticity.

### Atomic Force Microscopy (AFM)

All imaging experiments were carried out at room temperature in air with a Dimension 3000 AFM, Bruker. Images were acquired in the tapping mode with silicon (Si) cantilevers (spring constant of 20–100 N/m) and operated below their resonance frequency (typically 230–410 kHz). The images were flattened, and the contrast and brightness were adjusted for optimum viewing conditions. Amyloid samples were deposited on the surface of freshly cleaved mica (GoodFellow) for 5 min, washed 3 times with 200 *μ*l of DI water and dried in air at room temperature.

### Cell culture

SH-SY5Y (ATCC CRL-2266) were cultured in Dulbecco’s modified Eagle medium supplemented with 10%(v/v) FBS. Neural stem/progenitor cells were prepared from embryonic day 16.5 of rat embryos. Embryonic NSCs with Hes5-GFP and Dcx-RFP reporters were isolated at embryonic day 13.5 from a transgenic mouse carrying Hes5-GFP and Dcx-RFP by using established protocol [39, 40]. The resulting primary cells were verified to carry both Hes5-GFP and Dcx-RFP after isolation and allowed to grow for few passages before subjecting them to the experiments [41]. NSCs were cultured as neurospheres in culture media (DMEM/F12+ Glutamax (Gibco No:31331–028); 10 U/ml penicillin; 10 *μ*g/ml Streptomycin; B27 supplement (1:50); 0.02 *μ*g/ml FGF). As NSCs are sensitive to environmental variations, cell handling protocol before loading into the chip is examined systematically (including dissociation conditions and FACS sorting). To obtain the optimal results, NSCs spheres were collected and loaded into well plates at 24 hours after fresh dissociation, wherein each sphere contained ~7 to 10 cells. To avoid potential artifacts due to prolonged in vitro culture, only NSCs within 10 passages were used in the study. In control experiments, transferring chip-cultured NSCs to a well-plate showed the sphere-forming ability of Hes5-positive cells, validating Hes5 as a self-maintenance marker in our experiments [42].

### WST-1 cell viability assay

To evaluate cell viability, 10 *μ*l of WST-1 reagent was added per 100 *μ*l of cell culture and samples were incubated at 37°C for 4 h. The absorbance was measured using an ELISA plate reader (Labsystem Multiscan RC) at 450 nm. Cell viability was evaluated by normalizing the absorbance in wells containing cells treated with amyloid and/or oleic acid to the ones, where cells are maintained in culture medium for the same period of time. Values higher or lower than 100% represent cell proliferation and cell death, respectively. To ensure accurate machine reading, cells with only DMEM were set as the negative control, with Tris-HCL as the buffer control and cells treated with sphingosine as the positive control.

## Results

### OA micelles induced HEWL oligomerization and fibrillation

**Fig 1:**
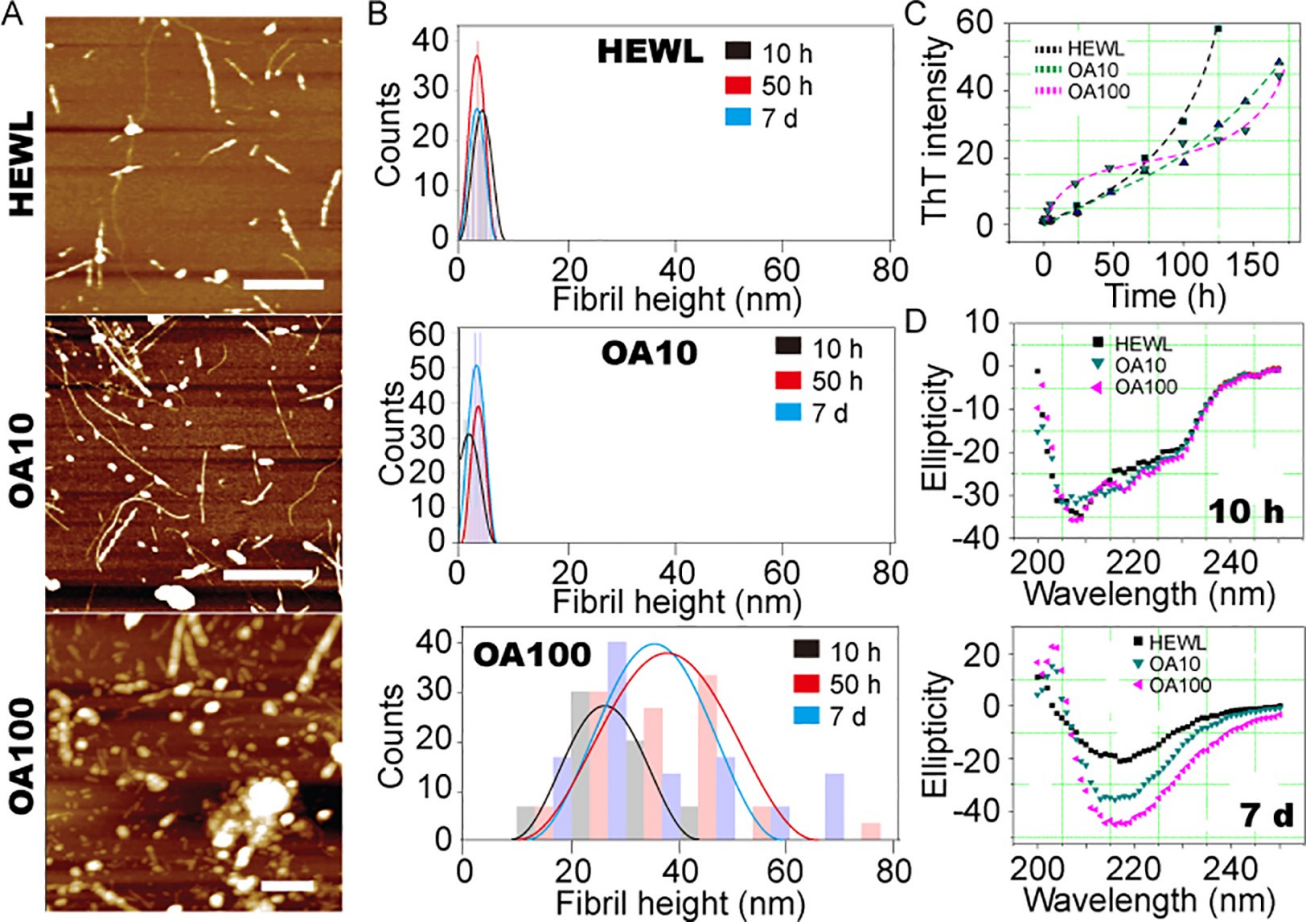
(A) AFM height image of 1.4 mM HEWL only (HEWL, top), 1.4 mM HEWL with 14 mM OA (OA10, middle), and 1.4 mM HEWL with 140 mM OA (OA100, down), after incubation for 1d. Scale bars denote 1000 nm in all images. (B) Height distribution of fibrils obtained by AFM measurement in samples containing only HEWL (HEWL, top), 1.4 mM HEWL with 14 mM OA (OA10, middle) and 1.4 mM HEWL with 140 mM OA (OA100, down). (C) Amyloid formation kinetics of 1.4 mM hen egg white lysozyme (HEWL) with 14mM and 140 mM OA (OA10 and OA100, respectively). (D) Far UV CD spectra of 1.4mM hen egg white lysozyme (HEWL) with OA. All samples are maintained in 20 mM glycine buffer under continuous shaking at 800 rpm, pH 2.3 and 57°C for up to7 days.

**Fig 2:**
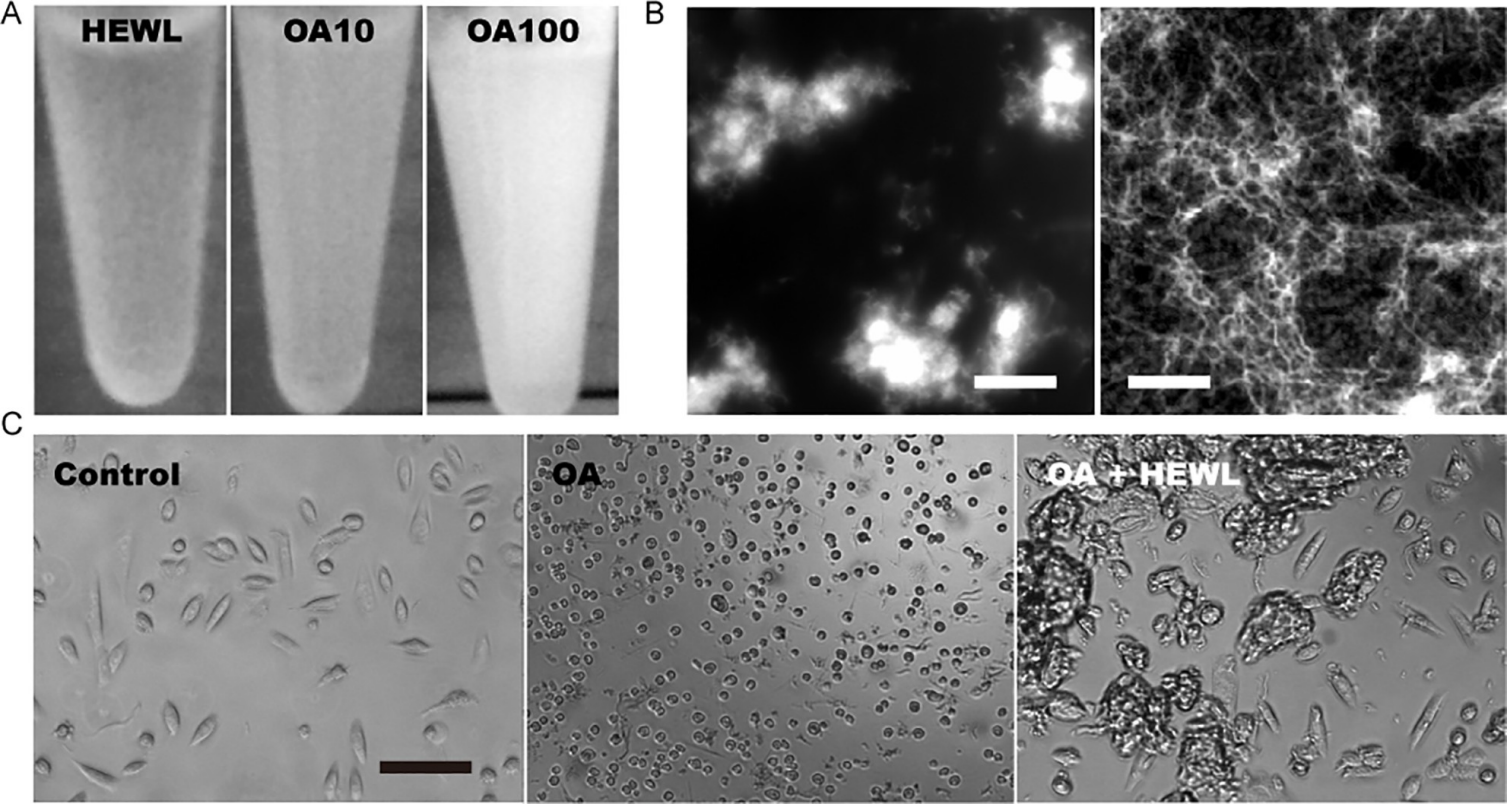
(A) Appearance of 1.4 mM HEWL (left), 1.4 mM HEWL with 14 mM OA (middle), and 1.4mM HEWL with 140 mM OA (right), after incubation in 20 mM glycine buffer under continuous shaking at 800 rpm, pH 2.3 and 57°C for 4 d. (B) Fluorescent microscopy images of pre-formed HEWL-OA clumps. Scale bares denote 100 *μ*m in the left panel, and 15 *μ*m in the right panel. (C) Optical images of SH-SY5Y Cells being maintained in DMEM culture medium (Control, left), culture medium containing 400 mM OA (OA,middle), and solution containing 4 *μ*M HEWL and 400 *μ*M OA (OA+HEWL, right). Scale bar denotes 100 *μ*M. The clumps were formed by positioning HEWL and OA mixture (HEWL 1.4 mM and OA 140 mM) under continuous shaking at 800 rpm and 57°C for 4 d.

HEWL amyloid formation with or without OA micelles was firstly examined by AFM (Fig 1A). To maintain micelle conformation throughout reaction, all samples were subjected to continuous shaking at 800 rpm, pH 2.3 and 57°C for various timespans (Fig 2A). HEWL fibrils sharing a similar height of ca. 5nm were produced after 1 d incubation. No measurable structural difference was observed upon prolonged incubation up to 7 d (Fig 1B). Rapid increase of ThT intensity indicates amyloidogenic nature of these fibrils, which is consistent with transitions in CD spectra (Figs 1C, S1A and S1B). At low OA doses, HEWL fibrils of ~5 nm in height and similar morphology were produced. Round-shaped HEWL aggregates (~5 nm in height) were emerged upon incubation with 14 mM OA (Fig 1A and 1B, middle row). In contrast, large quantities of round-shaped aggregates characterized by an AFM height of ~10 nm and diameters over 90 nm were observed upon incubation in the presence of 140 mM OA after the same duration. These oligomers further associate and form fibrillar structures. Large aggregates with heights ranging from 20 to 60 nm were also observed (Fig 1A and 1B, lower panel). However, an agitation, may not only promote HEWL amyloid formation, but also lead to dissipation of OA into small micelles (ca. 2 *μ*m in diameter). HEWL single molecules and oligomers may accumulate on OA micelles surface, which facilitates HEWL aggregations.

The kinetics of HEWL amyloid formation with and without the addition of OA was monitored using the ThT-binding assay and CD spectra (Fig 1C). The specific interaction of ThT with cross-*β*-sheet-containing amyloids leads to an increase in fluorescence emission. During the first 50 h of incubation, HEWL amyloid formation depended primarily on the concentration of OA. As manifested in ThT kinetics, the amyloid formation is a complex process consisting of a lag phase, during which intermediate oligomers were formed, and subsequently, amyloid fibrils emerged from oligomers as seeds, which is reflected in rapid growth phase [43]. OA micelles have shorten the lag-phase and facilitated amyloid formation. During prolonged incubation, ThT intensity of HEWL alone increased significantly higher compared to the HEWL complex with OA. A possible explanation is that OA micelles provide surfaces for HEWL molecule and oligomer aggregation, which stabilizes amyloidogenic oligomers and promotes their further growth. Further aggregation on micellar surfaces leads to lower detectable amyloid contents in solution. The transition in secondary structures during HEWL amyloid formation was monitored by far UV CD (Fig 1C). The CD spectrum of all amyloid samples developed after 7 days of incubation were characterized by a negative peak centered at 217 nm, which is typical for amyloid *β*-sheet structures. The *β*-sheet content, manifested in CD ellipticity, was proportional to the concentration of OA. The differences between ThT assay and CD spectrum may be due to the fact that CD spectroscopy measures the differential absorption of left- and right-handed circularly polarized transmitted light. Therefore, it is less sensitive to the effect of large amyloid aggregates.

### Effect of HEWL on OA cytotoxicity

**Fig 3:**
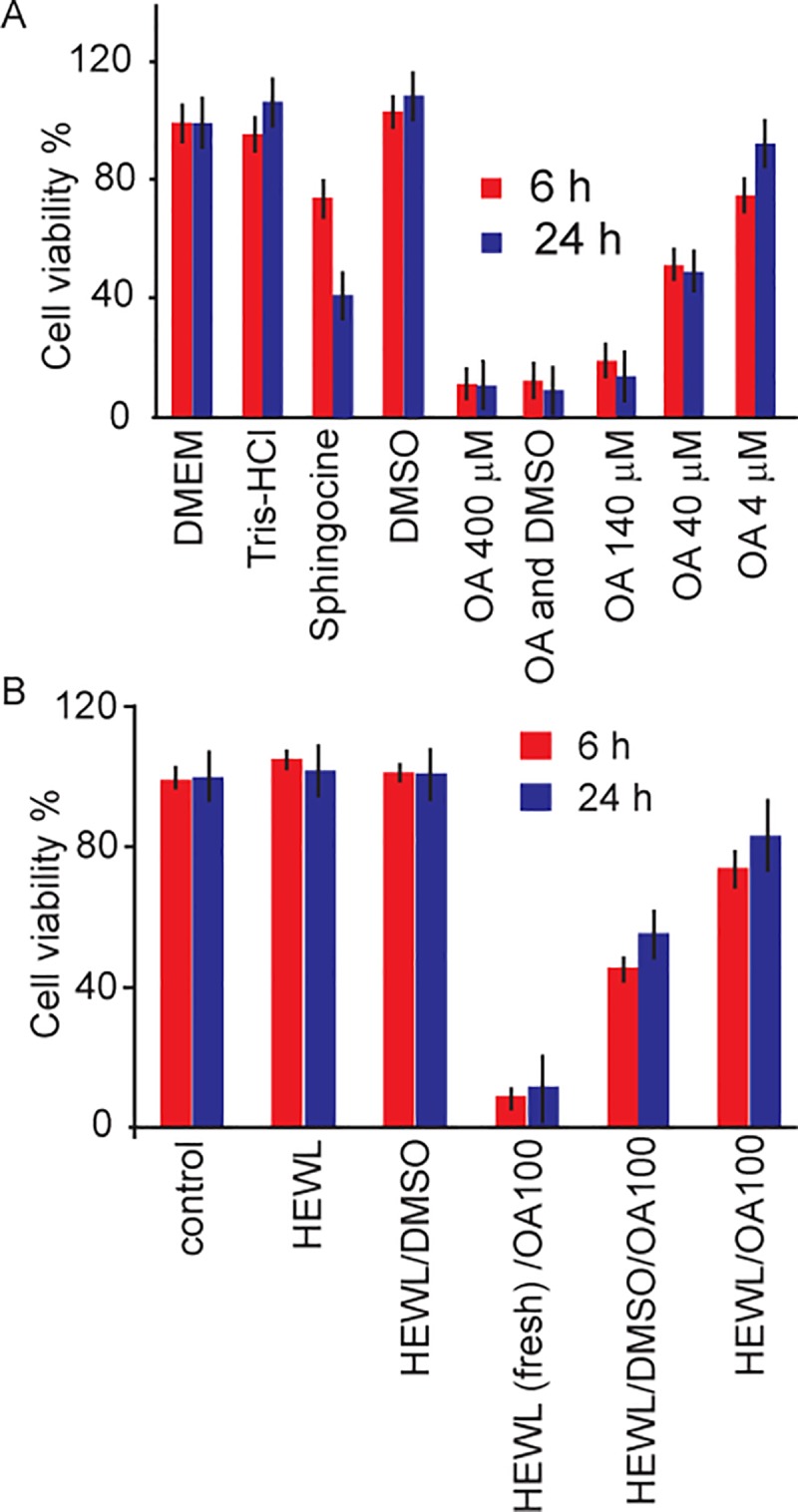
Measurements of SH-SY5Ycell viability by WST-1 assay in the presence of different concentrations of oleic acid (4 to 400 *μ*M), and (B) HEWL- OA complex. In control experiments the cells were incubated in DMEM cell culture buffer, Tris-HCl buffer, sphingocine and 16 mM DMSO. In DMEM culture medium, cell viability is equal to 100%. In the mixture of OA and DMSO, the OA concentration is 400 *μ*M and DMSO16 mM. All HEWL samples were prepared at HEWL concentration of 1.4 mM (OA is 14 and 140 mM) and aged 1 d in 20 mM glycine buffer under continuous shaking at800 rpm, pH 2.3 and 57°C.

**Fig 4:**
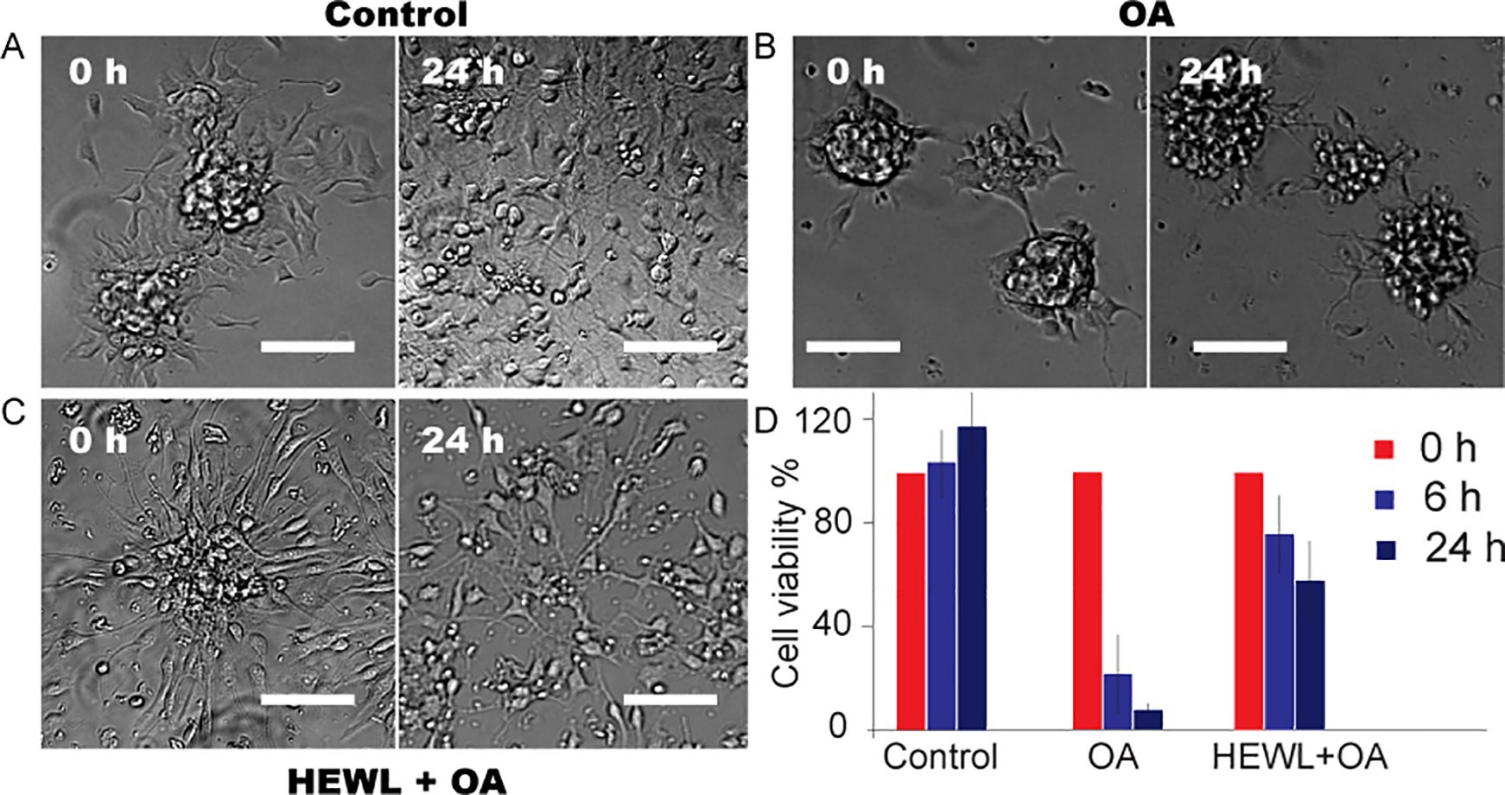
Brightfield images of neural stem cells (NSCs) exposed to (A) culture medium; (B) environmental conditions containing 400 *μ*M OA; (C) 1.4 mM HEWL with 140 mM OA. Scale bar denotes 100 *μ*m.The clumps were formed by positioning HEWL and OA mixture (HEWL 1.4 mM and OA140 mM)) under continuous shaking at 800 rpm and 57°C for 4 d. (D) Measurements of NSCs viability by WST-1 assay in the presence of culture medium, OA and HEWL-OA complex.

The effect of HEWL, OA micelles and their complexes on the viability of SH-SY5Y neuroblastoma cells and neural stem progenitor cells were assessed using the WST-1 assay and real-time microscopic monitoring. In viable cells, WST-1 undergoes reduction by mitochondrial dehydrogenases (succinate-tetrazolium reductase system) to soluble formazan, which serves as an indicator of the number of metabolically active cells. OA micelles were produced by incubating OA solution containing no HEWL under continuous shaking at 57°C 800 rpm for 1 day. The stock emulsion was then diluted using culture medium to desired OA concentrations before being transferred to 96-well plate for cell viability assessment. It is observed that SH-SY5Y cell viability decreased significantly at OA concentration as low as 4 *μ*M, and the toxicity of OA micelles is concentration-dependent (4 and 400 *μ*M OA correspond to 90% and 10% cell viability, respectively) (Fig 3A). DMSO, which is often used as a solvent for OA, is not toxic by itself. OA micelle neurotoxicity is further assessed using mouse neural stem progenitor cells (NSCs). NSCs cells were firstly cultured as neurospheres and transferred to 96-well plates for continuous imaging before adding OA and OA-HEWL complexes (Fig 4). Our results demonstrate that addition of 140 *μ*M OA induces quick NSCs cell death. As compared to control experiments, NSCs died before attached to lamin-coated surface, suggesting strong toxicity (Fig 4A, 4B and 4D).

OA micelles’ neurotoxicity is mediated by the addition of HEWL. When OA micelles are incubated with 1.4 mM HEWL for 1 d before cell treating, SH-SY5Y cell viability in the presence of 140 *μ*M OA increased from ~15% to ~80% (Fig 3B). Interestingly, freshly dissolved HEWL (without co-incubation) did not rescue SH-SY5Y cell from OA micelles, suggesting the protective role of HEWL amyloids but not of the native protein. The amyloidogenic nature of HEWL-OA complexes and their effect on SH-SY5Y cell were further investigated by using fluorescence microscopy. It is revealed that HEWL clumps were formed after co-incubation with OA micelles (Fig 2B). Using ThT as fluorophore, we demonstrate that HEWL aggregates were composed of large quantities of amyloid fibrils. Additionally, no SH-SY5Y cell death was observed even when they were in contact with HEWL-OA clumps, which were significantly different from samples containing only OA micelles (Fig 2C). Similar rescue effect of HEWL was observed in NSCs. The viability of primary neural stem and progenitor cells increased by more than 50% in the presence of HEWL. In contrast to control groups, where NSCs remained active during 24 h culture incubation, cells migrated at significantly lower speed, which was observed as cell positioning about their original locations as neurospheres (Fig 4C). Limited NSCs activities suggest that HEWL-OA complex clumps may affect cell plasticity.

## Discussion

Increasing evidence indicates that intracellular free fatty acids are incorporated into lipid droplets and transported by fatty acid binding proteins (FABPs) [44–46]. Fatty acids can then be released from membrane lipids to act as signaling molecules [47]. Fatty acid as micelles takes parts in a variety of biological activities, e.g. energy metabolism, signaling transduction and structural components synthesis or dissociation [48–50]. We herein studied effect of OA and HEWL-OA complex micelles on cells of neuronal origin, SH-SY5Y cell-line and primary mouse NSCs. It was revealed that OA micelles exert strong cell toxicity, while, HEWL amyloid formation can effectively mediate OA toxicity and rescue both cell types. Complexes of OA with amyloidogenic proteins (e.g. *α*-lactalbumin and equine lysozyme) have been extensively studied [51–54]. It has been demonstrated at the single molecular level that formation of protein-OA complexes dissembled OA micelles and affected cell viability. The structural and conformational differences of HEWL-OA complexes, which were produced under different experimental conditions, may account for our seemly contradictory results. Our hypothesis is that OA micelles, which were produced by continuous shaking, promoted HEWL amyloid formation by providing hydrophobic binding sites. HEWL accumulation surrounding OA micelles in turn, expedite formation of amyloidogenic sub-structures, which was observed as increase ThT fluorescence intensity at the initial stage (Fig 1C), and the formation of relatively large round-shaped oligomers (Fig 1A and 1B). These oligomers further accumulated and formed HEWL-OA clumps (Fig 2B). Buried amyloidogenic structures led to moderate increase in ThT intensity as compared to HEWL alone samples. In the meanwhile, submerged OA micelles lost their active binding sites, and that resulted in reduced neurotoxicity on SH-SY5Y and NSCs cells. To summarize, OA promoted HEWL aggregation and induced amyloid formation, which in turn consumed toxic OA species. Consequently, increased cell viability was induced by deceased free-diffused OA quantity.

## Conclusion

Our studies, for the first time, reveal that production of amyloidogenic structures, which is promoted by fatty acids micelles, may be a natural protective response. Compartmentalization of toxic species, i.e. amyloid protofibrils and fatty acids micelles, can be accompanied simultaneously by formation of senile plaques. Our studies on underlying mechanisms of amyloid formation may help to develop new therapeutic strategies.

## Supporting information

S1 Fig Far UV CD spectra of 10 and 20 mg/ml HEWL with DMSO and OA in 20 mM glycine buffer under continuous shaking at 800 rpm, pH 2.3 and 57°C. The spectra for different samples are drawn with solid lines to indicate the trend. (a, b) Changes of 10 and 20 mg/ml HEWL CD spectra with time, respectively; (c, d) CD spectra of 10 and 20 mg/ml HEWL respectively, with DMSO and OA after continuous shaking for 3 h; (e, CD spectra of 10 and 20 mg/ml HEWL respectively, with DMSO and OA after continuous shaking for 72 h. The CD spectra were recorded using 1 mm optical path length.
(DOCX)

S1 Video Real-time bright field imaging of NSCs maintained in solutions containing only culture medium (left), 400 *μ*M OA (middle), and 4 *μ*M HEWL and 400 *μ*M OA (right) for 24 hours. (AVI)
