# Correction: HEWL interacts with dissipated oleic acid micelles, and decreases oleic acid cytotoxicity

**DOI:** 10.1371/journal.pone.0221704

**Published:** 2019-08-22

**Authors:** Qin Huang, Dan Sun, Muhammad Zubair Hussain, Yonggang Liu, Ludmilla A. Morozova-Roche, Ce Zhang

There is an error in the XML that is causing the fifth author’s name, Ludmilla A. Morozova-Roche, to appear incorrectly in the citation. As a result, the fifth author’s name is indexed incorrectly. The correct initials are: Morozova-Roche LA. The correct citation is: Huang Q, Sun D, Zubair Hussain M, Liu Y, Morozova-Roche LA, Zhang C (2019) HEWL interacts with dissipated oleic acid micelles, and decreases oleic acid cytotoxicity. PLoS ONE 14(2): e0212648. https://doi.org/10.1371/journal.pone.0212648.

## References

[1] Huang Q, Sun D, Zubair Hussain M, Liu Y, A. Morozova-Roche L, Zhang C (2019) HEWL interacts with dissipated oleic acid micelles, and decreases oleic acid cytotoxicity. PLoS ONE 14(2): e0212648 10.1371/journal.pone.0212648 30794655 PMC6386356

