# Supplementary material for: HEWL interacts with dissipated oleic acid micelles, and decreases oleic acid cytotoxicity
Source: PLoS One. 2019 Feb 22;14(2):e0212648. doi: 10.1371/journal.pone.0212648 (PMC6386356; doi:10.1371/journal.pone.0212648)
Supplement: S1 Fig — The spectra for different samples are drawn with solid lines to indicate the trend. (a, b) Changes of 10 and 20 mg/ml HEWL CD spectra with time, respectively; (c, d) CD spectra of 10 and 20 mg/ml HEWL respectively, with DMSO and OA after continuous shaking for 3 h; (e, CD spectra of 10 and 20 mg/ml HEWL respectively, with DMSO and OA after continuous shaking for 72 h. The CD spectra were recorded using 1 mm optical path length. (DOCX) [file pone.0212648.s001.docx]

S1 Video. Real-time bright field imaging of NSCs maintained in solutions containing only culture medium (left), 400 μM OA (middle), and 4 μM HEWL and 400 μM OA (right) for 24 hours.


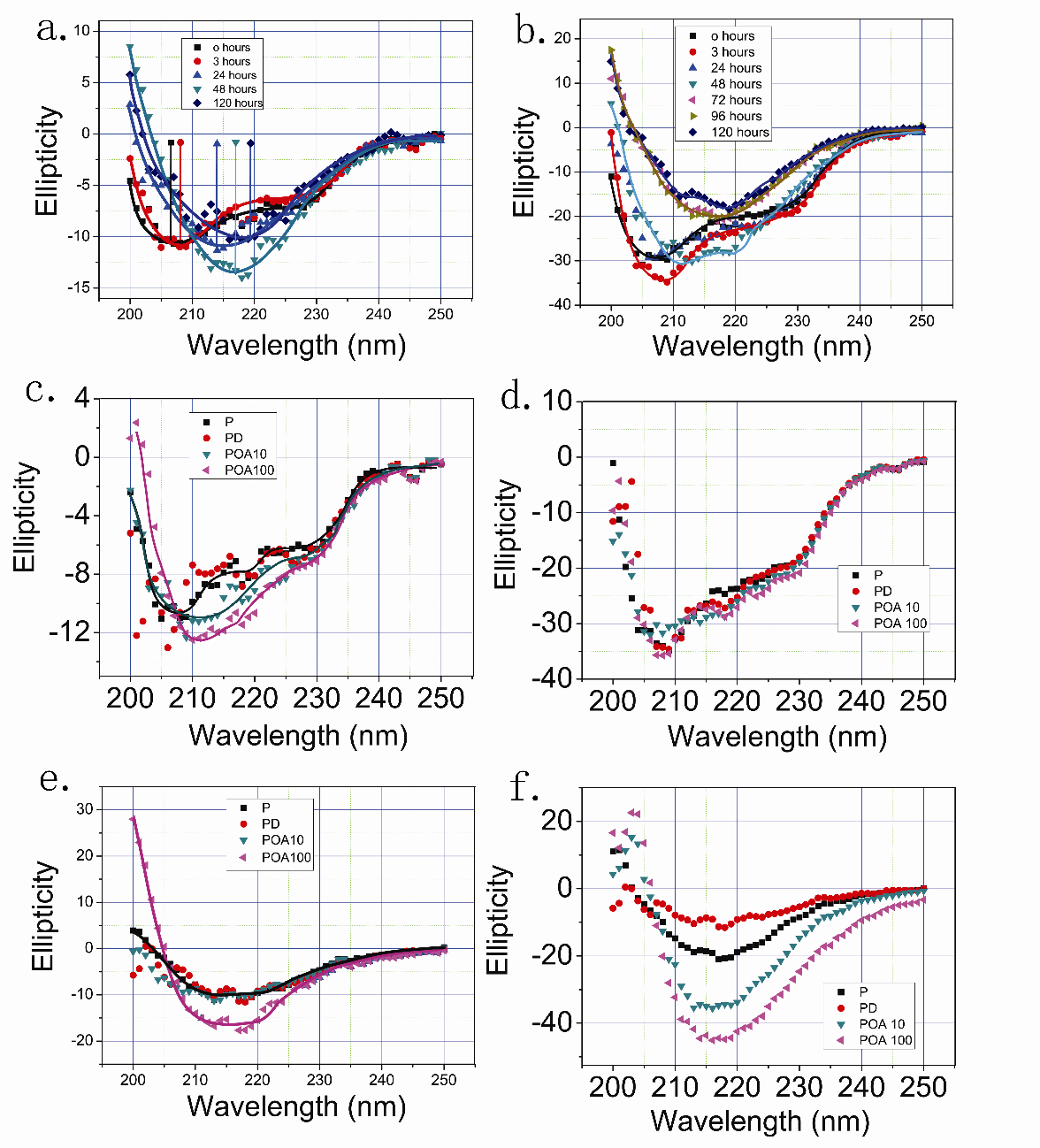


S1 Fig. Far UV CD spectra of 10 and 20 mg/ml HEWL with DMSO and OA in 20 mM glycine buffer under continuous shaking at 800 rpm, pH 2.3 and 57 °C. The spectra for different samples are drawn with solid lines to indicate the trend. (a, b) Evolution of 10 and 20 mg/ml HEWL with time respectively; (c, d) CD spectra of 10 and 20 mg/ml HEWL respectively, with DMSO and OA after continuous shaking for 3 h; (e, f) CD spectra of 10 and 20 mg/ml HEWL respectively, with DMSO and OA after continuous shaking for 72 h. The CD spectra were recorded with 1 mm optical path length.
